# Neighborhood disadvantage and individual-level life stressors in relation to breast cancer incidence in US Black women

**DOI:** 10.1186/s13058-021-01483-y

**Published:** 2021-11-22

**Authors:** Lauren E. Barber, Gary R. Zirpoli, Yvette C. Cozier, Lynn Rosenberg, Jessica L. Petrick, Kimberly A. Bertrand, Julie R. Palmer

**Affiliations:** 1 Department of Epidemiology, Boston University School of Public Health, Boston, MA USA; 2 Slone Epidemiology Center at Boston University, 72 East Concord Street, L-7, Boston, MA 02118 USA; 3 Department of Medicine, Boston University School of Medicine, Boston, MA USA

**Keywords:** Breast cancer, Disparities, Socioeconomic, Neighborhood, Estrogen receptor negative breast cancer

## Abstract

**Background:**

Research on psychosocial stress and risk of breast cancer has produced conflicting results. Few studies have assessed this relation by breast cancer subtype or specifically among Black women, who experience unique chronic stressors.

**Methods:**

We used prospective data from the Black Women’s Health Study, an ongoing cohort study of 59,000 US Black women, to assess neighborhood- and individual-level psychosocial factors in relation to risk of breast cancer. We used factor analysis to derive two neighborhood score variables after linking participant addresses to US Census data (2000 and 2010) on education, employment, income and poverty, female-headed households, and Black race for all households in each residential block group. We used Cox proportional hazards regression to estimate hazard ratios (HR) and 95% confidence intervals (CI) adjusted for established breast cancer risk factors.

**Results:**

During follow-up from 1995 to 2017, there were 2167 incident invasive breast cancer cases (1259 estrogen receptor positive (ER +); 687 ER negative (ER−)). For ER− breast cancer, HRs were 1.26 (95% CI 1.00–1.58) for women living in the highest quartile of neighborhood disadvantage relative to women in the lowest quartile, and 1.24 (95% CI 0.98–1.57) for lowest versus highest quartile of neighborhood socioeconomic status (SES). For ER+ breast cancer, living in the lowest quartile of neighborhood SES was associated with a reduced risk of ER+ breast cancer (HR = 0.83, 95% CI 0.70–0.98). With respect to individual-level factors, childhood sexual abuse (sexual assault ≥ 4 times vs. no abuse: HR = 1.35, 95% CI 1.01–1.79) and marital status (married/living together vs. single: HR = 1.29, 95% CI 1.08–1.53) were associated with higher risk of ER+, but not ER− breast cancer.

**Conclusion:**

Neighborhood disadvantage and lower neighborhood SES were associated with an approximately 25% increased risk of ER− breast cancer in this large cohort of Black women, even after control for multiple behaviors and lifestyle factors. Further research is need to understand the underlying reasons for these associations. Possible contributing factors are biologic responses to the chronic stress/distress experienced by individuals who reside in neighborhoods characterized by high levels of noise, crime and unemployment or the direct effects of environmental toxins.

**Supplementary Information:**

The online version contains supplementary material available at 10.1186/s13058-021-01483-y.

## Introduction

While recent overall breast cancer incidence rates in the US are similar for Black women and White women, US Black women are more likely to be diagnosed with estrogen receptor negative (ER−) breast cancer, which has fewer treatment options and a poorer prognosis [1]. Reasons for the disparity in incidence of ER− breast cancer have not been fully elucidated. Chronic psychosocial stress or distress is worth evaluating as a possible contributor, given that Black women experience high levels of psychosocial stress in multiple domains of life, including early life, employment, financial, relationships and community [2–4].

There have been numerous studies of psychosocial stress in relation to overall breast cancer risk. Some have reported associations of traumatic or stressful life events, including divorce/separation [5], death of a spouse [5], or death of a mother during childhood [6], to be associated with an elevated risk of breast cancer. Measures that may reflect chronic stress, such as social isolation [7], chronic major depression [6], and self-reported stress [8–10], have also been associated with breast cancer risk. However, there have been at least as many studies that have reported null [11–16] or inverse associations [17] of individual-level stress factors and breast cancer risk. Lower neighborhood-level socioeconomic status (SES) has also been linked to increased risk of breast cancer overall [18–21].

While differences in both study design and measurement of psychosocial stress may contribute to the inconsistent findings, failure to assess associations by ER status may also play a role. In studies that considered ER status separately, stressful experience, such as marital separation/divorce [22] and low neighborhood SES [21] were associated with an increased occurrence of ER− breast cancer. Stress can cause immune suppression, increased inflammation, epigenetic modifications, altered gene expression and changes in telomere length [23, 24], pathways that may be involved in the development of ER− breast cancer. In addition, three case-only studies found that the relative proportion of ER− breast cancer and/or triple negative (ER−, progesterone receptor negative, HER2 negative) breast cancer was higher among women residing in lower SES neighborhoods than among women residing in higher SES neighborhoods [21, 25–27].

We assessed the relation of individual-level and neighborhood-level psychosocial stress factors with risk of ER+, ER− and triple negative breast cancer in over 20 years of follow-up data from Black women participating in a prospective cohort study.

## Methods

### Study population

In 1995, 59,000 women aged 21 to 69 years enrolled in the Black Women’s Health Study (BWHS), a prospective cohort study of women from across the US who self-identified as Black [28, 29]. At baseline and every two years afterwards, participants have completed a self-administered questionnaire. Follow-up is complete for 85% of person-years. Women were excluded from the present analysis if they had received a cancer diagnosis prior to 1995. This left 57,442 BWHS participants available for analysis. The Boston University Institutional Review Board approved the study protocol.

### Exposure ascertainment

#### Individual-level early-life factors

On the 2017 questionnaire, participants were asked whether they had experienced loss of a parent or guardian (mother, father, guardian) before the age of 18. They were also asked if, during their childhood or adolescence, a member of their household served time in prison. The 2013 questionnaire ascertained data on childhood financial hardship. Participants were classified as having severe financial hardship in childhood if their household did not have enough money for food/housing and received public assistance/welfare; as having moderate hardship if their answer was yes to one question, and as having no hardship if their answer was no to both questions. Experiences of sexual and physical abuse in childhood and adolescence were reported in 2005. For each time period (childhood or adolescence), exposure was classified as no abuse, someone exposed their genitals to the participant but no sexual assault, sexual assault 1–3 times, sexual assault ≥ 4 times or physical abuse only. We repeated the analyses using a reference group of no to both childhood and adolescent abuse. Since results were similar to the primary analyses, we do not present results from analyses using the modified reference group.

#### Individual-level adult-life factors

Marital status, reported in 1995 and on two follow-up questionnaires, was categorized as single, married/living together, separated/divorced or widowed. Educational attainment was reported in 1995 and 2003, and grouped as ≤ 12, 13–15, ≥ 16 years of education. Ascertainment of perceived experiences of daily and institutional racism have been described previously [30]. In 1997, participants were asked how often they experienced the following due to their race: receiving poor service at restaurants or stores, being treated as if you are not intelligent, people acting as if they are afraid of you, being treated as if you are dishonest and people acting as if they are better than you. Response choices were: “Never,” “A few times a year,” “Once a month,” “Once a week” or “Almost every day.” Responses were given a value of 1–5. Values were summed and divided by five to create a daily racism score ranging from 1 to 5, with 1 representing never. The score was categorized into quartiles. Regarding institutional racism, participants reported whether they had been treated unfairly due to their race with regard to employment, housing or the police. We classified participants as reporting no to all domains, yes to 1, yes to 2, or yes to all 3.

Depression was measured in 1999 and 2005 using the 20-item Center for Epidemiological Studies Depression (CESD) scale [31], which asked respondents to describe how frequently they have experienced depressive symptoms in the past week on a four-point scale. Responses were assigned a value of 0–3. Values were summed across all 20 items, with higher scores indicating more depressive symptoms. Scores were categorized into three levels (0–15, 16–22, ≥ 23) based on clinical recommendations [32] and prior research [33]. Perceived stress was measured on the 2005 questionnaire using a 4-item Perceived Stress Scale [34], which characterizes the frequency of stressful feelings during the past month. Answer choices were given a score of 0–4 and summed, with scores of 0–4, 5–8 and ≥ 9 classified as low, average and high perceived stress, respectively.

#### Neighborhood-level factors

Assessment of neighborhood SES has been described elsewhere [35]. In brief, neighborhood SES was determined by linking participants’ geocoded addresses at each questionnaire cycle to year 2000 (for questionnaire years 1995–2003) and 2010 (for subsequent years) US Census data at the block group level. Factor analysis was performed to create a neighborhood SES score based on six variables (median household income; median housing value; percentage of households receiving interest, dividend or net rental income; percentage of adults aged ≥ 25 years who have completed college; percentage of employed persons aged ≥ 16 years who are in occupations classified as managerial, executive or professional; and percentage of families with children that are not headed by a single female), with higher values indicating higher SES. The score was categorized into quartiles.

To determine neighborhood concentrated disadvantage, we linked participants’ addresses from 1995 to 2003 questionnaires to year 2000 Census data, addresses from 2005 to 2009 questionnaires to years 2005–2009 American Community Survey (ACS) data and addresses from 2011 to 2015 questionnaires to years 2007–2011 ACS data at the block group level. We conducted a factor analysis of six variables as described in prior literature [36]: percentage of individuals below the poverty line, percentage of individuals on public assistance, percentage of female headed households, percentage unemployed, percentage of individuals below age 18 and percentage of Black residents. Factor scoring coefficients from each factor analysis were used to weight the six variables. The variables were summed to create a neighborhood concentrated disadvantage score and categorized into quartiles. Distributions of each of the factors that contribute to the neighborhood SES and neighborhood concentrated disadvantage scores, overall and in the lowest and highest quartiles of each score, are displayed in Additional file 1: Table S1.

We evaluated the joint effect of neighborhood SES and concentrated disadvantage by creating a combination variable with the following categories: highest SES and least disadvantaged, intermediate SES and intermediate disadvantage, lowest SES and most disadvantaged. In addition, percentage of Black residents, which can be a measure of social cohesion, was assessed in quartiles with data obtained through linkage to Census and ACS data.

### Outcome ascertainment

Incident invasive breast cancer was the outcome of interest. Breast cancer cases were identified through self-report on follow-up questionnaires, linkage to the National Death Index, and linkage to the 24 state cancer registries in which 95% of the participants lived. Trained study personnel blinded to exposure status reviewed pathology reports and registry data to confirm diagnoses and collect information on diagnosis date and tumor characteristics. ER, progesterone receptor (PR) and human epidermal growth factor receptor 2 (HER2) status were available for 90% of breast cancer cases.

### Statistical analysis

Cox proportional hazards regression was used to calculate hazard ratios and 95% confidence intervals (CI) for the association of each individual- and neighborhood-level stressor with risk of breast cancer, according to ER status and for triple negative breast cancer. We stratified regression models by questionnaire cycle and age in one-year increments, such that age was the underlying timescale. Participants accrued person-time beginning at the time of exposure assessment and ending at breast cancer diagnosis, death or end of the study period in 2017, whichever occurred first. Follow-up was considered to begin in 1995 for most exposures, in 1997 for daily and institutional racism, in 1999 for CESD score and in 2005 for perceived stress.

Because study participants lived in almost all 50 states across the US, there was very little clustering within neighborhood block groups. 57% percent of block groups had only one BWHS participant, 19% had only two and 9% had only three. Nevertheless, to account for clustering within neighborhoods, we performed a sensitivity analysis using generalized estimating equations to assess associations between neighborhood-level factors and breast cancer risk. The estimates of relative risk from this analysis were nearly identical to those from the primary analysis and therefore we present results from the standard Cox proportional hazards models.

The following factors were included in multivariable regression models: age at menarche (≤ 11, 12–13, ≥ 14 years), BMI at age 18 (< 20, 20–24, ≥ 25 kg/m^2^), parity (nulliparous, 1, 2, ≥ 3 births), age at first birth (< 20, 20–24, ≥ 25 years), breastfeeding (never, ever), menopausal status (premenopausal, postmenopausal), age at menopause (< 45, 45–49, ≥ 50 years), oral contraceptive use (never, < 1, 1–4, ≥ 5 years), first-degree family history of breast cancer (yes, no), geographic region (Northeast, South, Midwest, West) and vigorous physical activity (none, < 5, ≥ 5 h/week). Cigarette smoking, waist-hip ratio, Alternative Healthy Eating Index, and type 2 diabetes were also considered, but were not included in the final regression models because their inclusion did not appreciably change the HRs and they are not established breast cancer risk factors. Individual-level stressors were considered as confounders in analyses of neighborhood-level factors, but their inclusion did not change effect estimates. Missing indicator terms were used to account for missing covariate data. Time-varying covariates were updated at each questionnaire cycle.

We investigated associations of neighborhood-level factors across strata of age (< 50 vs. ≥ 50 years) and education (≤ 12 vs. > 12 years). In education-stratified analyses, we collapsed quartiles 3 and 4 of neighborhood SES and quartiles 1 and 2 of neighborhood concentrated disadvantage because of small numbers in some of the categories.

Linear trend was assessed in analyses of neighborhood-level exposures by treating the measures as ordinal variables. Reported *p* values are two-sided with a 0.05 level of significance. Analyses were performed using SAS 9.4 (Cary, North Carolina).

## Results

During the study period from 1995 to 2017, there were 2167 incident invasive breast cancer cases; 1259 were classified as ER+, 687 as ER− and 310 as triple negative.

The distribution of individual- and neighborhood-level factors at baseline is displayed in Table 1. The median age was 38, range 21–69. Approximately 25% of the study participants were widowed, separated or divorced, 19% had ≤ 12 years of education, and 29% lived in the lowest SES and most disadvantaged neighborhoods. Approximately 24% of participants had experienced death of a parent or guardian before age 18 and 18% had experienced sexual abuse in childhood. In addition, 11% of participants reported experiencing institutional racism in all three spheres queried, 29% had a CESD score of ≥ 16 and 13% reported a high level of perceived stress.

**Table 1:** Participant characteristics at baseline or at first assessment of each exposure and number of incident breast cancer cases

	N	
Age in years (1995)	57,442	Median = 38
		%
Body mass index (kg/m^2^) (1995)		
< 25	21,426	37.3
25–29	17,489	30.4
30–34	9176	16.0
≥ 35	7606	13.2
Geographic region (1995)		
Northeast	15,719	27.4
South	17,677	30.8
Midwest	13,369	23.3
West	10,677	18.6
Marital status (1995)		
Single	19,726	34.7
Married or living together	22,652	39.8
Separated, divorced, widowed	14,473	25.5
Years of education (1995)		
12 years or less	11,012	19.2
13–15 years	20,686	36.1
≥ 16 years	25,631	44.7
Death of a parent or caregiver before age 18 (2017)	7776	24.0
Member of household served time in prison before age 18 (2017)	2455	7.2
Any childhood sexual abuse (2005)	6387	17.9
Any adolescent sexual abuse (2005)	6904	19.4
Severe childhood financial hardship (2013)	4163	12.0
Neighborhood SES and concentrated disadvantage joint variable (1995)		
Highest SES, least disadvantaged	15,929	30.2
Intermediate SES/disadvantaged	21,425	40.7
Lowest SES, most disadvantaged	15,337	29.1
Institutional racism (1997)		
No to all 3 spheres (employment, housing, police)	14,664	30.4
Yes to 1	16,142	33.4
Yes to 2	12,037	24.9
Yes to 3	5446	11.3
CESD Scale (Depression scale based on 20 questions) (1999)		
Score 0–15	27,977	71.3
Score 16–22	6015	15.3
Score ≥ 23	5270	13.4
Perceived Stress Scale (2005)		
Score 0–4, low perceived stress	16,148	48.2
Score 5–8, average perceived stress	12,873	38.4
Score ≥ 9, high perceived stress	4513	13.5
Incident breast cancer cases (1995 though 2017)	2167	
ER+ breast cancer	1259	
ER− breast cancer	687	
Triple negative breast cancer	310	

*SES* socioeconomic status, *CESD* center for epidemiological studies depression

### Individual-level factors

None of the individual-level factors were associated with risk of ER− or triple negative breast cancer (Table 2). However, for ER+ breast cancer, positive associations were observed with childhood sexual abuse (sexual assault ≥ 4 times vs. no abuse: HR = 1.35, 95% CI 1.01–1.79) and marital status (married/living together vs. single: HR = 1.29, 95% CI 1.08–1.53). The HR for ≥ 16 vs. ≤ 12 years of education was 1.39 (95% CI 1.19–1.64) in age-adjusted analyses, but reduced to 1.18 (95% CI 1.00–1.41) with control for parity, age at first birth and other established risk factors.

**Table 2:** Association between individual-level psychosocial stress factors and risk of breast cancer by ER status and for triple negative breast cancer

Exposure	ER+			ER−			Triple negative		
	Cases	Age-adjusted HR (95% CI)^a^	MV-adjusted HR (95% CI)^b^	Cases	Age-adjusted HR (95% CI)^a^	MV-adjusted HR (95% CI)^b^	Cases	Age-adjusted HR (95% CI)^a^	MV-adjusted HR (95% CI)^b^
*Early-life stressors*									
Loss of a parent/guardian before age 18									
No	554	1.00 (ref)	1.00 (ref)	251	1.00 (ref)	1.00 (ref)	128	1.00 (ref)	1.00 (ref)
Death of mother	69	1.14 (0.89–1.47)	1.19 (0.92–1.53)	28	1.08 (0.73–1.59)	1.09 (0.73–1.61)	17	1.29 (0.78–2.15)	1.29 (0.77–2.14)
Death of father/guardian	102	0.85 (0.69–1.05)	0.87 (0.71–1.08)	45	0.84 (0.61–1.15)	0.84 (0.61–1.16)	26	0.94 (0.62–1.44)	0.95 (0.62–1.45)
Member of household served time in prison before age 18									
No	732	1.00 (ref)	1.00 (ref)	318	1.00 (ref)	1.00 (ref)	164	1.00 (ref)	1.00 (ref)
Yes	39	0.75 (0.54–1.03)	0.78 (0.57–1.08)	27	1.07 (0.73–1.55)	1.18 (0.79–1.75)	14	1.16 (0.67–2.01)	1.16 (0.67–2.01)
Childhood financial hardship									
None	585	1.00 (ref)	1.00 (ref)	261	1.00 (ref)	1.00 (ref)	138	1.00 (ref)	1.00 (ref)
Moderate	176	0.98 (0.83–1.16)	1.02 (0.86–1.20)	80	0.97 (0.76–1.25)	0.98 (0.76–1.26)	36	0.82 (0.57–1.19)	0.83 (0.57–1.20)
Severe	73	0.79 (0.62–1.01)	0.84 (0.66–1.08)	37	0.87 (0.61–1.23)	0.87 (0.61–1.23)	24	1.07 (0.69–1.65)	1.10 (0.71–1.71)
Childhood sexual abuse									
No abuse	426	1.00 (ref)	1.00 (ref)	223	1.00 (ref)	1.00 (ref)	113	1.00 (ref)	1.00 (ref)
Exposed to genitals of an adult	56	1.06 (0.80–1.40)	1.04 (0.79–1.38)	33	1.16 (0.80–1.68)	1.12 (0.78–1.62)	23	1.60 (1.02–2.50)	1.56 (0.99–2.45)
Sexual assault 1–3 times	83	0.90 (0.71–1.14)	0.91 (0.72–1.15)	46	0.91 (0.66–1.26)	0.89 (0.65–1.22)	25	0.99 (0.64–1.53)	0.96 (0.62–1.49)
Sexual assault ≥ 4 times	55	1.29 (0.97–1.71)	1.35 (1.01–1.79)	19	0.80 (0.50–1.28)	0.77 (0.48–1.24)	11	0.93 (0.50–1.73)	0.90 (0.48–1.67)
Physical abuse only	239	1.13 (0.96–1.32)	1.12 (0.95–1.31)	120	1.05 (0.84–1.31)	1.04 (0.83–1.30)	64	1.12 (0.82–1.52)	1.11 (0.81–1.51)
Adolescent sexual abuse									
No abuse	514	1.00 (ref)	1.00 (ref)	273	1.00 (ref)	1.00 (ref)	140	1.00 (ref)	1.00 (ref)
Exposed to genitals of an adult	63	1.07 (0.83–1.40)	1.06 (0.82–1.38)	26	0.81 (0.54–1.22)	0.80 (0.53–1.20)	12	0.73 (0.41–1.33)	0.72 (0.40–1.29)
Sexual assault 1–3 times	130	1.07 (0.88–1.30)	1.11 (0.91–1.35)	70	1.02 (0.78–1.32)	0.99 (0.75–1.29)	41	1.16 (0.82–1.65)	1.13 (0.79–1.61)
Sexual assault ≥ 4 times	31	1.08 (0.75–1.55)	1.16 (0.80–1.67)	13	0.81 (0.46–1.42)	0.80 (0.46–1.40)	10	1.23 (0.65–2.35)	1.23 (0.64–2.34)
Physical abuse only	121	1.06 (0.87–1.29)	1.08 (0.89–1.32)	59	0.93 (0.70–1.24)	0.93 (0.70–1.23)	33	1.02 (0.70–1.49)	1.02 (0.70–1.50)
*Adult-life stressors*									
Marital status									
Single	216	1.00 (ref)	1.00 (ref)	132	1.00 (ref)	1.00 (ref)	58	1.00 (ref)	1.00 (ref)
Married/live together	605	1.25 (1.07–1.47)	1.29 (1.08–1.53)	317	1.17 (0.95–1.45)	0.96 (0.77–1.21)	139	0.88 (0.64–1.21)	1.08 (0.77–1.53)
Separated/divorced	340	1.07 (0.89–1.28)	1.12 (0.93–1.36)	192	1.11 (0.88–1.40)	0.94 (0.73–1.20)	95	1.05 (0.81–1.36)	1.06 (0.81–1.38)
Widowed	94	1.11 (0.86–1.45)	1.21 (0.92–1.58)	45	1.15 (0.80–1.67)	0.99 (0.68–1.45)	18	0.85 (0.51–1.43)	0.89 (0.53–1.49)
Years of education									
≤ 12 years	194	1.00 (ref)	1.00 (ref)	121	1.00 (ref)	1.00 (ref)	58	1.00 (ref)	1.00 (ref)
13–15 years	355	1.17 (0.98–1.40)	1.10 (0.92–1.32)	213	1.07 (0.85–1.34)	1.03 (0.82–1.29)	96	0.98 (0.71–1.36)	0.92 (0.66–1.28)
≥ 16 years	707	1.39 (1.19–1.64)	1.18 (1.00–1.41)	353	1.09 (0.88–1.34)	1.04 (0.83–1.31)	156	0.94 (0.69–1.27)	0.89 (0.64–1.24)
Daily racism									
Quartile 1	206	1.00 (ref)	1.00 (ref)	112	1.00 (ref)	1.00 (ref)	60	1.00 (ref)	1.00 (ref)
Quartile 2	321	1.12 (0.94–1.33)	1.10 (0.92–1.31)	171	1.04 (0.82–1.33)	1.02 (0.81–1.30)	88	1.00 (0.72–1.39)	0.99 (0.71–1.38)
Quartile 3	285	1.06 (0.88–1.27)	1.04 (0.87–1.25)	147	0.94 (0.73–1.20)	0.93 (0.72–1.19)	80	0.94 (0.67–1.32)	0.94 (0.67–1.32)
Quartile 4	247	1.13 (0.94–1.37)	1.14 (0.94–1.38)	110	0.85 (0.65–1.11)	0.85 (0.65–1.11)	55	0.79 (0.54–1.14)	0.78 (0.54–1.14)
Institutional racism									
No to all	302	1.00 (ref)	1.00 (ref)	162	1.00 (ref)	1.00 (ref)	86	1.00 (ref)	1.00 (ref)
Yes to 1	331	1.00 (0.85–1.17)	0.98 (0.84–1.14)	176	0.98 (0.79–1.21)	0.96 (0.78–1.19)	95	1.00 (0.74–1.33)	0.98 (0.73–1.32)
Yes to 2	267	1.01 (0.86–1.19)	0.99 (0.84–1.17)	120	0.84 (0.66–1.06)	0.82 (0.65–1.05)	62	0.82 (0.59–1.13)	0.80 (0.58–1.11)
Yes to 3	132	1.08 (0.88–1.33)	1.07 (0.87–1.31)	62	0.93 (0.70–1.25)	0.94 (0.70–1.26)	30	0.84 (0.56–1.28)	0.83 (0.55–1.26)
CESD Scale									
Score 0–15	678	1.00 (ref)	1.00 (ref)	324	1.00 (ref)	1.00 (ref)	184	1.00 (ref)	1.00 (ref)
Score 16–22	124	1.01 (0.83–1.22)	1.02 (0.84–1.24)	60	0.98 (0.74–1.29)	0.99 (0.75–1.31)	34	0.98 (0.68–1.42)	0.99 (0.69–1.43)
Score ≥ 23	87	0.80 (0.64–1.00)	0.84 (0.67–1.05)	45	0.83 (0.61–1.13)	0.85 (0.62–1.17)	25	0.82 (0.54–1.24)	0.83 (0.54–1.27)
Perceived stress Scale									
Score 0–4 low perceived stress	277	1.00 (ref)	1.00 (ref)	135	1.00 (ref)	1.00 (ref)	116	1.00 (ref)	1.00 (ref)
Score 5–8, average perceived stress	214	1.02 (0.86–1.23)	1.05 (0.87–1.25)	101	0.99 (0.76–1.28)	1.01 (0.78–1.31)	101	1.18 (0.90–1.54)	1.19 (0.91–1.56)
Score ≥ 9, high perceived stress	51	0.75 (0.55–1.01)	0.77 (0.57–1.05)	22	0.65 (0.41–1.03)	0.65 (0.41–1.03)	20	0.73 (0.46–1.18)	0.72 (0.45–1.17)

*ER* estrogen receptor, *HR* hazard ratio, *CI* confidence interval, *MV* multivariable, *SES* socioeconomic status

^a^Hazard ratios adjusted for age and time period

^b^Hazard Ratios additionally adjusted for age at menarche, BMI at age 18, parity, age at first birth, breastfeeding, menopausal status, age at menopause, oral contraceptive use for 5 + years, family history of breast cancer, geographic region and physical activity

### Neighborhood-level factors

Women who lived in the lowest quartile of neighborhood SES were estimated to have a 24% (HR = 1.24, 95% CI 0.98–1.57) increased risk of ER− breast cancer compared to those living in the highest quartile (Table 3). Similarly the HR for highest vs. lowest quartile of neighborhood concentrated disadvantage was 1.26 (95% CI 1.00–1.58). The HR for the joint association of low neighborhood SES and neighborhood disadvantage with ER− breast cancer risk was 1.23 (95% CI 1.00–1.52; *P*_trend_ = 0.05). The HR for highest versus lowest quartile of percentage of Black residents was 1.21 (95% CI 0.95–1.53; data not shown). However, with additional control for neighborhood SES, the HR was reduced to 1.14 (95% CI 0.87–1.48) and was also similar when additional control was made for neighborhood disadvantage instead of neighborhood SES. Hazard ratios for triple negative breast cancer were similar to those for ER− cancer, but less precise. In contrast to results for ER− breast cancer, women who lived in the lowest quartile of neighborhood SES were estimated to have a reduced risk of ER+ breast cancer (HR = 0.83, 95% CI 0.70–0.98). Neighborhood concentrated disadvantage was not associated with ER+ disease.

**Table 3:** Association between neighborhood-level factors and risk of breast cancer by ER status and for triple negative breast cancer

Exposure	ER+			ER−			Triple negative		
	Cases	Age-adjusted HR (95% CI)^a^	MV-adjusted HR (95% CI)^b^	Cases	Age-adjusted HR (95% CI)^a^	MV-adjusted HR (95% CI)^b^	Cases	Age-adjusted HR (95% CI)^a^	MV-adjusted HR (95% CI)^b^
Neighborhood SES									
Quartile 1 (low)	245	0.73 (0.62–0.86)	0.83 (0.70–0.98)	163	1.17 (0.94–1.47)	1.24 (0.98–1.57)	65	1.29 (0.91–1.84)	1.29 (0.91–1.84)
Quartile 2	282	0.86 (0.73–1.01)	0.93 (0.79–1.10)	159	1.16 (0.92–1.45)	1.20 (0.96–1.51)	68	1.20 (0.85–1.71)	1.20 (0.85–1.71)
Quartile 3	300	0.87 (0.74–1.02)	0.91 (0.78–1.06)	159	1.10 (0.88–1.38)	1.12 (0.89–1.40)	81	1.14 (0.81–1.60)	1.14 (0.81–1.60)
Quartile 4 (high)	336	1.00 (ref)	1.00 (ref)	143	1.00 (ref)	1.00 (ref)	75	1.00 (ref)	1.00 (ref)
P-trend		0.0003	0.05		0.14	0.06		0.46	0.15
Neighborhood concentrated disadvantage									
Quartile 1 (low)	303	1.00 (ref)	1.00 (ref)	142	1.00 (ref)	1.00 (ref)	71	1.00 (ref)	1.00 (ref)
Quartile 2	306	0.98 (0.84–1.15)	1.02 (0.87–1.19)	161	1.11 (0.89–1.40)	1.14 (0.91–1.43)	69	1.01 (0.72–1.42)	1.04 (0.74–1.46)
Quartile 3	315	0.99 (0.85–1.16)	1.05 (0.89–1.23)	165	1.14 (0.91–1.43)	1.18 (0.94–1.48)	72	1.19 (0.86–1.65)	1.25 (0.90–1.74)
Quartile 4 (high)	261	0.83 (0.70–0.97)	0.92 (0.77–1.09)	170	1.19 (0.95–1.49)	1.26 (1.00–1.58)	63	1.13 (0.81–1.58)	1.25 (0.88–1.76)
P-trend		0.03	0.47		0.13	0.06		0.32	0.13
Neighborhood SES and concentrated disadvantage combination									
Highest SES, least disadvantaged	379	1.00 (ref)	1.00 (ref)	176	1.00 (ref)	1.00 (ref)	79	1.00 (ref)	1.00 (ref)
Intermediate SES/disadvantage	508	0.97 (0.85–1.11)	1.03 (0.90–1.17)	266	1.11 (0.92–1.35)	1.14 (0.94–1.38)	122	1.12 (0.84–1.49)	1.16 (0.87–1.54)
Lowest SES, most disadvantaged	304	0.80 (0.69–0.93)	0.90 (0.77–1.05)	199	1.17 (0.96–1.44)	1.23 (1.00–1.52)	89	1.14 (0.84–1.55)	1.25 (0.91–1.72)
P-trend		0.005	0.21		0.12	0.05		0.39	0.16

*ER* estrogen receptor, *HR* hazard ratio, *CI* confidence interval, *MV* multivariable, *SES* socioeconomic status

^a^Hazard ratios adjusted for age and time period

^b^Hazard Ratios additionally adjusted for age at menarche, BMI at age 18, parity, age at first birth, breastfeeding, menopausal status, age at menopause, oral contraceptive use for 5 + years, family history of breast cancer, geographic region and physical activity

As shown in Table 4, in analyses stratified on educational status (a measure of individual SES), an association of residence in disadvantaged neighborhoods with increased risk of ER− breast cancer was present only among women with ≤ 12 years of education (HR = 1.94, 95% CI 1.26–2.99) and not among women with at least some college education. For low neighborhood SES, there was evidence of a positive association within both strata of educational status. Associations were not modified by age (data not shown).

**Table 4:** Association between neighborhood-level factors and risk of ER− breast cancer stratified by years of education

Exposure	≤ 12 years of education				> 12 years of education			
	Cases	Person-years	Age-adjusted HR (95% CI)^a^	MV-adjusted HR (95% CI)^b^	Cases	Person-years	Age-adjusted HR (95% CI)^a^	MV-adjusted HR (95% CI)^b^
Neighborhood SES^c^								
Quartile 1 (low)	51	75,846	1.32 (0.86–2.03)	1.48 (0.95–2.31)	112	182,610	1.09 (0.88–1.37)	1.12 (0.89–1.40)
Quartile 2	36	52,647	1.31 (0.82–2.09)	1.39 (0.87–2.22)	123	205,192	1.08 (0.87–1.33)	1.09 (0.88–1.36)
Quartiles 3 and 4 (higher)	35	65,509	1.00 (ref)	1.00 (ref)	267	476,572	1.00 (ref)	1.00 (ref)
Neighborhood concentrated disadvantage^d^								
Quartiles 1 and 2 (lower)	36	72,687	1.00 (ref)	1.00 (ref)	267	469,971	1.00 (ref)	1.00 (ref)
Quartile 3	30	53,516	1.15 (0.71–1.86)	1.23 (0.75–2.00)	135	217,510	1.08 (0.88–1.33)	1.09 (0.88–1.34)
Quartile 4 (high)	59	72,780	1.67 (1.10–2.53)	1.94 (1.26–2.99)	111	189,808	1.01 (0.81–1.26)	1.02 (0.81–1.28)

*ER* estrogen receptor, *HR* hazard ratio, *CI* confidence interval, *MV* multivariable, *SES* socioeconomic status

^a^Hazard ratios adjusted for age and time period

^b^Hazard ratios additionally adjusted for age at menarche, BMI at age 18, parity, age at first birth, breastfeeding, menopausal status, age at menopause, oral contraceptive use for 5 + years, family history of breast cancer, geographic region and physical activity

^c^*P*_interaction_ = 0.91

^d^*P*_interaction_ = 0.15

## Discussion

In this large prospective cohort of US Black women, residence in disadvantaged or low SES neighborhoods was associated with an approximately 25% increased risk of ER− breast cancer. Similar results were observed for triple negative breast cancer, based on smaller numbers. Our findings are consistent with prior studies, most of which were case-only or registry-based [20, 21, 25–27, 37]. Those studies reported that hormone receptor negative breast cancer was more common than hormone receptor positive cancer among women living in areas characterized by low neighborhood SES [26, 27, 37], neighborhood poverty [25] and neighborhood deprivation [20], or who were born in states classified as “Jim Crow” states before passage of the US Civil Rights Act of 1964 [21]. However, one other study observed no association of neighborhood SES with ER− breast cancer [38].

The association between neighborhood disadvantage and ER− breast cancer was apparent only among women with a high school education or less. It may be that higher educational attainment (or factors associated with higher educational attainment) acts as a buffer against effects of an adverse neighborhood environment. A similar interaction was reported in a case-only study [26].

Contrary to the results for ER− breast cancer, low neighborhood SES was associated with a reduced risk of ER+breast cancer rather than an increased risk. Two previous studies have reported similar associations with ER+[20] or hormone receptor-positive [38] cancer. In two other studies, there was no association [38, 39] and in a third, the association disappeared after adjustment for breast cancer risk factors [35]. Additionally, in studies that considered all breast cancers together, living in higher SES neighborhoods was associated with elevated risk [39–42]. Historically, women who have low SES themselves and live in lower SES neighborhoods have tended to have higher parity and earlier age at first birth, factors that are associated with reduced risk of ER+ breast cancer. Controlling for these two reproductive factors did indeed reduce the magnitude of the association of neighborhood SES with risk of ER+ breast cancer (HR from 0.73 to 0.83 and p-trend from 0.003 to 0.05); residual confounding by these and other reproductive factors may explain the remaining association.

We also examined percentage of Black residents in a neighborhood, as it has been a marker of social cohesion. Two case-only studies of neighborhood racial composition suggested an association between percentage of Black residents in a neighborhood and breast cancer subtypes, independent of other neighborhood-level factors [26, 27]. In the present analysis, we observed a positive association between the highest quartile of percent Black residents and risk of ER− breast cancer. However, the association was attenuated with control for neighborhood SES or concentrated disadvantage.

Our findings suggest that where an individual lives matters with respect to development of ER− breast cancer. Furthermore, the fact that the associations were unchanged by adjustment for multiple factors, including diet, exercise, BMI and type 2 diabetes, indicates that the impact of neighborhood characteristics is not simply due to individual characteristics and behaviors shared by residents of certain neighborhoods. Associations with increased risk of ER− breast cancer may be driven by unmeasured aspects of the neighborhood environment such as environmental toxins, noise and lack of safety. Neighborhoods of lower SES are more often characterized by social (crime, conflict, drug use), physical (abandoned buildings, vandalism, noise, disrepair) and environmental (air pollution, toxins, chemicals) disadvantage [24, 43]. They are often marked by higher rates of poverty and greater disorder [43]. Consequently, living in disadvantaged and low SES neighborhoods can induce fear [43], precipitate unhealthy behaviors and diet, and increase exposure to environmental, physiological and psychological stressors, resulting in chronic stress [24]. Previous research has found a higher allostatic load, a measure of the biological dysregulation of physiological systems due to repeated stress [44], in Black vs. White women [45], especially among Black women from disadvantaged [46] and low SES neighborhoods [47]. Black Americans, particularly Black women, are more likely than Whites to live in impoverished neighborhoods, regardless of their education or income level [48, 49], potentially increasing exposure to chronic stress.

Research on the biological impacts of chronic stress suggests that stress may influence breast cancer through multiple pathways. Stress activates the sympathetic nervous system and hypothalamic–pituitary–adrenal axis, which in turn release hormones that trigger the fight or flight response to combat the perceived threat [50]. This process is called allostasis [50]. Chronic stress can produce a prolonged state of allostasis, leading to an increase in stress hormones (e.g., glucocorticoids) [50], whose downstream effects may influence breast cancer development [24]. For example, increases in stress hormones can reduce immune activity and increase levels of pro-inflammatory molecules [24, 50] shown to be involved in breast carcinogenesis [51]. Stress-induced inflammation may also cause epigenetic alterations, such as aberrant DNA methylation [24, 44], including in genes that may be involved in breast cancer development. Finally, stress may indirectly impact breast cancer by increasing the prevalence of breast cancer risk factors, such as obesity [52] and type 2 diabetes [53].

Despite the biologic plausibility of a relation between chronic psychosocial stress and increased risk of breast cancer, or specifically of ER− breast cancer, we found little or no evidence of association with individual-level factors. The only individual-level finding supporting a stress/breast cancer risk hypothesis was the increased risk of ER+ breast cancer (but not ER−) for women who had experienced sexual assault during childhood. One prior study found that women who reported sexual abuse any time during the lifetime had a twofold increased risk of breast cancer [54]. Biological mechanisms through which childhood sexual abuse may influence breast cancer risk, and risk of ER+ breast cancer in particular, are unclear. More research is needed to replicate these findings and shed light on potential mechanisms. In addition, given that we evaluated a number of other individual-level factors, the results concerning sexual abuse and ER+ breast cancer could be a chance finding. We found no evidence of an increased risk of breast cancer for widowed or divorced women relative to single, never married women, as was reported in two previous studies [5, 55]. Finally, having a greater number of years of education was associated with a small increase in risk of ER+ breast cancer, similar to what has been reported in most [35, 40, 41, 56, 57], but not all [26, 39], prior studies of the topic and may be due to incomplete control of confounding by factors such as age at first birth, growth velocity, and childhood diet, each of which is correlated with higher SES status.

The major limitation with regard to analyses of individual-level factors was a lack of information on how much these ostensible stressors actually caused distress in each individual. Experiences of encountering stressors (e.g., loss of spouse, loss of a parent, frequent experiences of racism) combined with how the experiences affect the individual person will give a measure of psychosocial “distress”, which is probably more relevant biologically. Individuals respond differently to stressful situations and similar situations can take a very different toll. We did not have a measure of distress, such as would be obtained from responses to a PTSD assessment tool. Misclassification of the actual distress resulting from various factors will have reduced our power to detect associations with individual factors even if chronic psychosocial distress plays a role in the etiology of breast cancer.

Other limitations of the study include small numbers for analyses stratified by educational level and for overall analyses of triple negative breast cancer. There is not a gold standard for measuring neighborhood SES or neighborhood concentrated disadvantage, but the composite score variables we used were similar if not identical to composite variables used by other investigators. Although we controlled for many individual-level confounders, we did not have detailed data on additional neighborhood characteristics such as exposure to environmental toxins and thus could not evaluate whether or to what extent those exposures explained the observed association with neighborhood disadvantage.

A major strength of the study was the cohort study design, which enabled estimation of relative risk of ER− breast cancer rather than a comparison of ER− with ER+ breast cancer, as in previous case-only studies. Detailed data on breast cancer risk factors, including a complete reproductive history, permitted control for these factors, something that was not possible in registry-based studies. The sample size was large, enabling assessment of associations by ER status and triple negative breast cancer. Importantly, we had collected data on participant address every two years and had geocoded the addresses, allowing us to create time-varying neighborhood-level factors for disadvantage, SES, and segregation.

## Conclusions

Black women living in disadvantaged neighborhoods were estimated to have an increased risk of ER− breast cancer compared to those living in more advantaged neighborhoods. Given the high prevalence of living in disadvantaged neighborhoods among Black women, it is possible that the disproportionately high incidence of ER− breast cancer in US Black women may be partially explained by neighborhood environment.

## Supplementary Information

**Additional file 1: Table S1.** Distributions of the factors that contribute to neighborhood SES and neighborhood concentrated disadvantage scores, at baseline in 1995.

## Data Availability

Information regarding BWHS data availability is available at http://www.bu.edu/bwhs/for-researchers/ (or by email: bwhs@bu.edu).
