# Supplementary material for: Neighborhood disadvantage and individual-level life stressors in relation to breast cancer incidence in US Black women
Source: Breast Cancer Res. 2021 Nov 22;23:108. doi: 10.1186/s13058-021-01483-y (PMC8609879; doi:10.1186/s13058-021-01483-y)
Supplement: Supplementary file 1 — Additional file 1: Table S1. Distributions of the factors that contribute to neighborhood SES and neighborhood concentrated disadvantage scores, at baseline in 1995. [file 13058_2021_1483_MOESM1_ESM.docx]

| **Supplementary Table 1. Distributions of the factors that contribute to neighborhood SES and neighborhood concentrated disadvantage scores, at baseline in 1995.** | | | | |
| --- | --- | --- | --- | --- |
| **Factors** | **Entire analytic sample**  **Mean (std dev)** |  | **Quartile 1 (low)**  **Mean (std dev)** | **Quartile 4 (high)**  **Mean (std dev)** |
| Neighborhood SES |  |  |  |  |
| Median household income | $43,418.8 (20,638.2) |  | $23,037.6 (7,065.1) | $68,470.7 (20,068.8) |
| Median housing value | $139,479.6 (101,377.4) |  | $95,741.4 (87,367.4) | $215,498.4 (123,355.7) |
| Percentage of households receiving interest, dividend, or net rental income | 26.0 (16.0) |  | 10.1 (5.4) | 47.3 (11.8) |
| Percentage of adults aged ≥25 years who have completed college | 23.8 (17.9) |  | 8.7 (5.9) | 45.2 (16.4) |
| Percentage of employed persons aged ≥16 years who are in occupations classified as managerial, executive, or professional | 60.2 (15.3) |  | 47.0 (11.8) | 76.3 (9.9) |
| Percentage of families with children that are not headed by a single female | 77.4 (14.9) |  | 59.8 (12.8) | 91.5 (5.7) |
| Neighborhood concentrated disadvantage |  |  |  |  |
| Percentage of individuals below the poverty line | 16.4 (13.5) |  | 4.9 (3.6) | 33.4 (12.7) |
| Percentage of individuals on public assistance | 6.1 (6.8) |  | 1.2 (1.4) | 14.4 (7.9) |
| Percentage of female headed households | 39.6 (14.5) |  | 24.7 (9.9) | 54.2 (10.7) |
| Percentage unemployed | 5.2 (3.9) |  | 2.3 (1.5) | 9.1 (4.7) |
| Percentage of individuals below age 18 | 26.6 (8.3) |  | 23.5 (7.9) | 32.4 (7.7) |
| Percentage of Black residents | 49.1 (34.9) |  | 14.4 (17.6) | 75.6 (25.0) |
| SES, Socioeconomic status; Std dev, Standard deviation. | | | | |
